# Gemcitabine: immunomodulatory or immunosuppressive role in the tumor microenvironment

**DOI:** 10.3389/fimmu.2025.1536428

**Published:** 2025-04-09

**Authors:** Mahnaz Nemati, Chou-Yi Hsu, Deepak Nathiya, M. Ravi Kumar, Enwa Felix Oghenemaro, Muthena Kariem, Parjinder Kaur, Deepak Bhanot, Ahmed Hjazi, Tayebeh Azam Saedi

**Affiliations:** ^1^ Amir Oncology Hospital, Shiraz University of Medical Sciences, Shiraz, Iran; ^2^ Thunderbird School of Global Management, Arizona State University, Phoenix, AZ, United States; ^3^ Department of Pharmacy Practice, NIMS Institute of Pharmacy, NIMS University Rajasthan, Jaipur, India; ^4^ Department of Basic Science & Humanities, Raghu Engineering College, Visakhapatnam, India; ^5^ Department of Pharmaceutical Microbiology, Faculty of Pharmacy, Delta State University, Abraka, Delta State, Nigeria; ^6^ Department of Medical Analysis, Medical Laboratory Technique College, The Islamic University, Najaf, Iraq; ^7^ Department of Medical Analysis, Medical Laboratory Technique College, The Islamic University of Al Diwaniyah, Al Diwaniyah, Iraq; ^8^ Department of Medical Analysis, Medical Laboratory Technique College, The Islamic University of Babylon, Babylon, Iraq; ^9^ Chandigarh Pharmacy College, Chandigarh Group of Colleges-Jhanjeri, Mohali, Punjab, India; ^10^ Centre for Research Impact & Outcome, Chitkara University Institute of Engineering and Technology, Chitkara University, Rajpura, Punjab, India; ^11^ Department of Medical Laboratory, College of Applied Medical Sciences, Prince Sattam bin Abdulaziz University, Al-Kharj, Saudi Arabia; ^12^ Department of Genetics, Faculty of Science, Islamic Azad University, Tonekabon, Iran

**Keywords:** gemcitabine, cancer, immunomodulatory, immunosuppressive, immunotherapy

## Abstract

Gemcitabine (GEM), a nucleoside analog chemotherapy agent, has been widely used in the treatment of various cancers. In recent years, there has been growing interest in understanding the immunomodulatory or immunosuppressive effects of GEM. The immunomodulatory roles of GEM could influence the anti-tumor immune responses via several mechanisms, such as modulation of antigen presentation, cytokine production, and immune cell population. Furthermore, there is evidence that GEM enhances the therapeutic efficacy of immunotherapies, including oncolytic viruses, immune checkpoint inhibitors, CAR T-cells, and therapeutic vaccines. On the other hand, accumulating evidence also proposed that GEM may act as an immunosuppressive agent within the tumor microenvironment, resulting in immune evasion of tumor cells and tumor growth. These paradoxical roles of GEM in modifying immune responses highlight the complexity of GEM interaction with immune cells and responses within the tumor microenvironment. This review aims to provide an overview of the immunomodulatory and immunosuppressive effects of GEM within the tumor microenvironment and how GEM affects the efficacy of cancer immunotherapy.

## 1 Introduction

Gemcitabine (GEM), a deoxycytidine nucleoside analog (2′-deoxy-2′,2′-difluorocytidine; dFdC), has emerged as a cornerstone in the treatment of various types of cancer due to its potent cytotoxic activities and manageable safety profile (1, 2). Initially approved for the treatment of pancreatic cancer in 1997, GEM has since shown efficacy in a wide range of malignancies, including bladder cancer, breast cancer, ovarian cancer, and lung cancer (1). The mechanism of action of GEM involves inhibition of DNA synthesis by targeting ribonucleotide reductase, leading to cell cycle arrest and apoptosis (3). Despite its widespread use, challenges such as drug resistance and toxicities remain significant hurdles in optimizing GEM-based therapies.

Beyond its direct cytotoxic effects on cancer cells, emerging evidence suggests that GEM may also modulate immune responses within the tumor microenvironment (TME), thereby influencing the anti-tumor immune response (4). The interplay between GEM and the immune system has garnered increasing interest in recent years, as immunotherapy continues to revolutionize cancer treatment paradigms. Understanding the complex interactions between GEM and immune responses and GEM’s role as immunomodulatory and/or immunosuppressive within the TME holds significant promise for enhancing therapeutic outcomes and overcoming resistance mechanisms in cancer patients (5, 6). This review aims to elucidate the impact of GEM on immune responses and its combination with immunotherapeutic agents, including oncolytic viruses, immune checkpoint inhibitors, chimeric antigen receptor (CAR) T-cells, and therapeutic vaccines. By exploring the relationship between GEM and the immune system, as an immunomodulatory and/or immunosuppressive agent, this review provides insights to optimize cancer therapy using GEM.

## 2 Gemcitabine and its mechanisms of action

**Figure 1:**
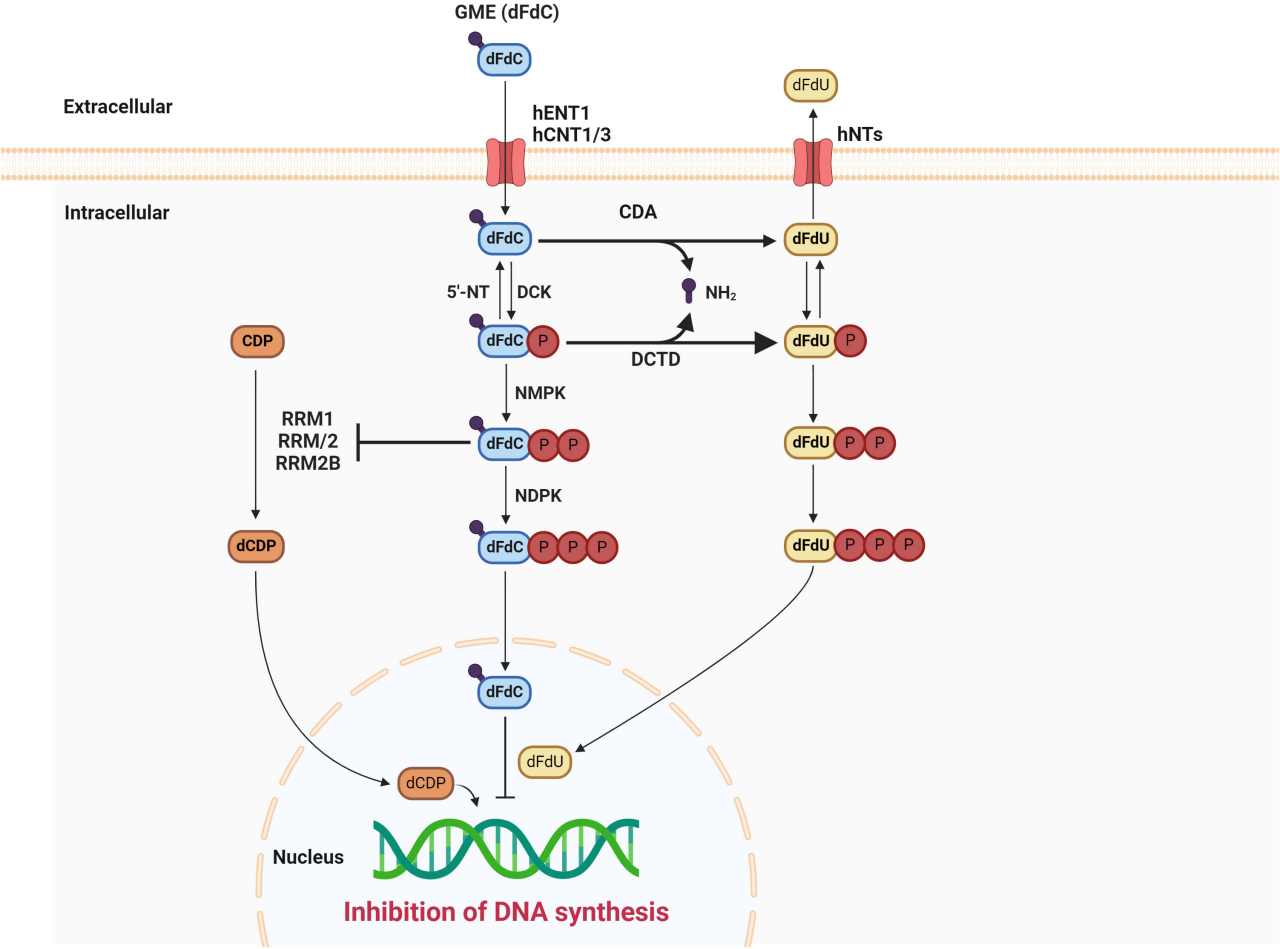
The mechanism of action of gemcitabine. DCK, deoxycytidine kinase; 5′-NT, 5′-nucleotidase; NMPK, nucleoside monophosphate kinase; NDPK, nucleoside diphosphate kinase; CDA, cytidine deaminase; DCTD, deoxycytidylate deaminase; dFdU, 2’2’ difluorodeoxyuridine; CDP, cytidinediphosphate; dCDP, deoxycytidine diphosphate; RRM1, ribonucleotide reductase subunit M1; RRM2, ribonucleotide reductase subunit M2.

GEM, a hydrophilic molecule, necessitates using nucleoside transporters, specifically human concentrative nucleoside transporters (hCNTs) and human equilibrative nucleoside transporters (hENTs), to traverse the cellular lipid bilayer. Uptake into cells is primarily mediated by hENT1, although hENT2, hCNT1, hCNT2, and hCNT3 also contribute to this process (7, 8). Once intracellular, the prodrug GEM undergoes a three-step phosphorylation cascade initiated by deoxycytidine kinase (dCK), forming GEM monophosphate (dFdCMP). Subsequent phosphorylation steps yield the active diphosphate (dFdCDP) and triphosphate (dFdCTP) forms (1). GEM’s primary intracellular metabolite, dFdCTP, functions as a competitive inhibitor of deoxycytidine triphosphate (dCTP) in DNA synthesis. This competitive action enables dFdCTP incorporation into the growing DNA chain, ultimately halting DNA elongation and inducing apoptotic cell death. A unique mechanism known as “masked chain termination” secures GEM’s position within the DNA molecule. Here, dFdCTP is integrated at the DNA strand’s terminus. Subsequent nucleotide addition prevents further DNA polymerase activity, while proofreading exonucleases are incapable of removing the GEM nucleotide from this final position (9). **Figure 1** summarizes the mechanism of action of GEM.

## 3 Gemcitabine and cancer immunotherapy agents

**Figure 2:**
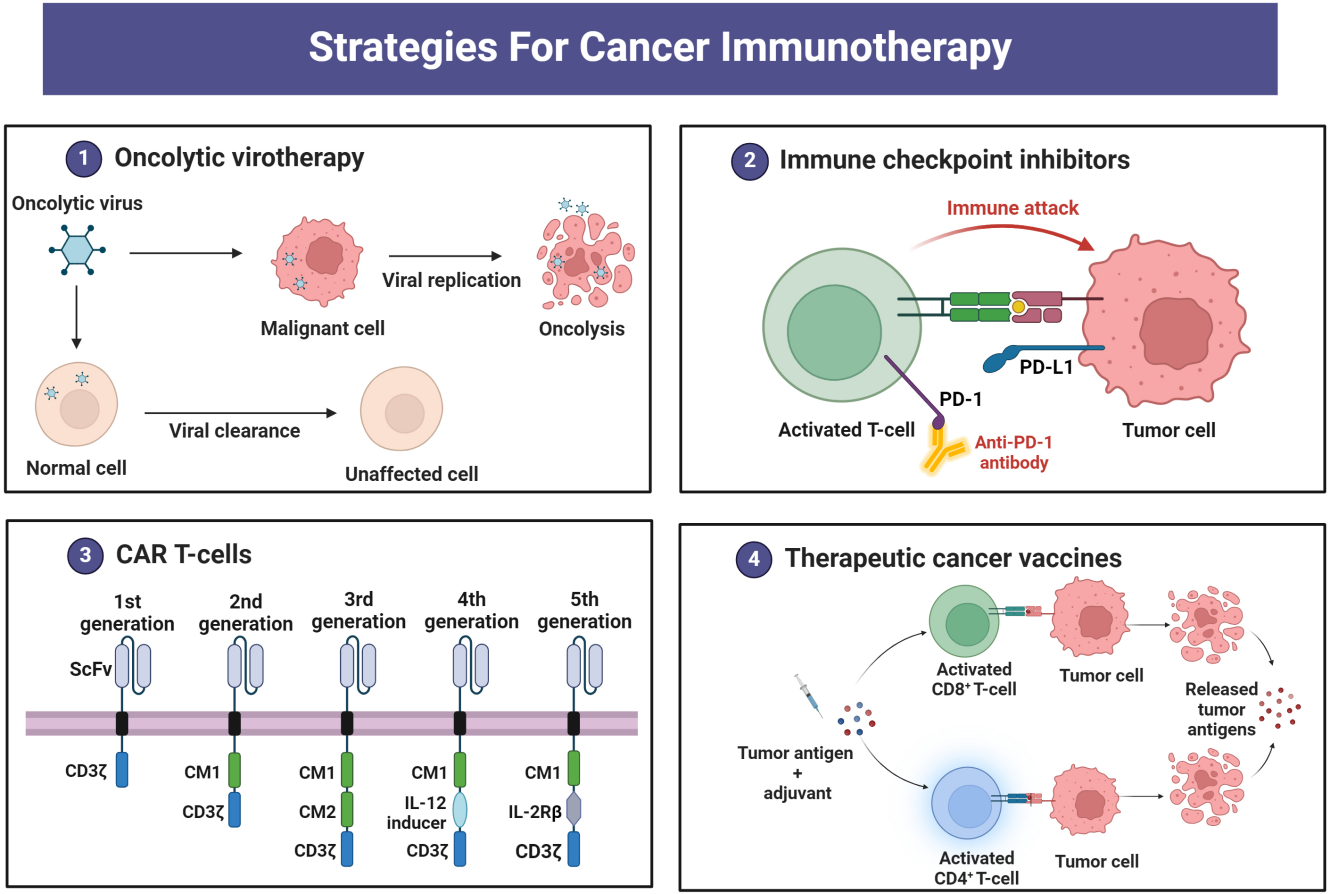
Immunotherapy strategies for cancer treatment.

There are various strategies in the treatment of cancer, based on eliciting immune responses, called cancer immunotherapy. **Figure 2** depicts four main cancer immunotherapy strategies, including oncolytic viruses, immune checkpoint inhibitors, CAR T-cells, and therapeutic vaccines. **Table 1** summarizes the chemoimmunotherapy application of GEM in combination with other therapeutic agents in clinical trials.

**Table 1:** The chemoimmunotherapy application of GEM in clinical trials.

GEM dose	Combination with	Cancer	Phase	Identifier
1000mg per square meter	RT, Cisplatin, Penpulimab	NPC	II	NCT06788002
1000mg per square meter	RT, Tadalafil	PC	I	NCT01903083
1000mg per square meter	Tislelizumab, Cisplatin, Trilaciclib	UC	III	NCT06364904
1000mg per square meter	Trastuzumab, Cisplatin, Nivolumab	BTC	I/II	NCT05749900
NA	Cemiplimab, RT	NSCLC	II	NCT06623656
1000mg per square meter	HT, RT, Cisplatin, Durvalumab	BTC	I	NCT06546969
NA	Sargramostim, Peptide vaccine, Capecitabine	PC	III	NCT00425360
NA	BCG vaccine	NMIBC	I/II	NCT04179162
1000mg per square meter	Cisplatin, Adebrelimab, CCRT	NPC	II	NCT06455410
NA	Adebrelimab, RT, Chemotherapy	NSCLC	II	NCT06424899

RT, radiotherapy; NPC, nasopharyngeal cancinoma; PC, pancreatic cancer; UC, urothelial carcinoma; BTC, biliary tract cancer; NA, not available; NSCLC, non-small cell lung cancer; HT, hyperthermia; BCG, Bacillus Calmette-Guérin; NMIBC, non-muscle invasive bladder cancer; CCRT, concurrent chemoradiotherapy; NPC, nasopharyngeal carcinoma.

### 3.1 Gemcitabine and oncolytic viruses

Oncolytic viruses (OVs) are viruses that preferentially replicate within tumor cells and lyse them, without any harmful effects on normal cells (10, 11). They can naturally kill tumor cells, such as Newcastle disease virus (NDV), vaccinia virus (VV), measles virus (MV), poliovirus, and reovirus, or modification in their genomic structure directs them to be oncolytic, such as herpes simplex virus-1 (HSV-1) and adenovirus (Ad) (12). OVs exert their anti-tumor activities not only through the destruction of malignant cells, but also they elicit immune responses against the released pathogen-associated molecular patterns (PAMPs), damage-associated molecular patterns (DAMPs), and tumor-associated antigens (TAAs) (13, 14). Efforts in this field led to the FDA approval of an HSV-based OV, called Talimogene laherparepvec (T-VEC, Imlygic), against melanoma. Moreover, three others have been approved for the regional use, including Rigvir in Latvia, Oncorine (H101) in China, and DELYTACT in Japan (15, 16).

The combination of chemotherapy and viruses, called chemovirotherapy, is one of the effective therapeutic strategies in cancer treatment. For example, Angelova et al. showed that combining GEM and H-1PV not only reduces GEM dosage without adding general toxicity, but also prevents metastases, reduces tumor growth, and prolongs survival in a pancreatic cancer model when H1-PV was administered as a second-line regimen after GEM (17). The synergistic anti-tumor effects of GEM in combination with other OVs also were reported, such as its combination with HF10 in colorectal cancer (CRC) (18), oncolytic VV in pancreatic cancer (19, 20), oncolytic measles virus in pancreatic cancer (21), and oncolytic Ad in pancreatic cancer (22) and lung cancer (23). Mechanistically, GEM exhibits suppressive activity against myeloid-derived suppressor cells (MDSCs) in the spleen, which inhibits T-cell responses, while preserving the populations of NK cells, B-cells, CD4^+^ T-cells, and CD8^+^ T-cells. Increases in the levels of IFN-γ following GEM + OV administration also eliminate tumor-associated macrophages (TAMs) which stimulate tumor growth (18). Moreover, the replication of OVs in tumor cells and their lysis breaks weak penetration of GEM into the tumor parenchyma, facilitating GEM penetration (19). There is evidence that a relaxin-encoding oncolytic Ad, YDC002, overcomes extracellular matrix (ECM)-dependent resistance to GEM in pancreatic cancer by degrading ECM components, such as elastin, fibronectin, and collagens, leading to enhanced diffusion of GEM (24). Additionally, the synergistic anti-tumor activity of GEM + OVs could be due to their convergence in activating apoptosis pathways (20, 25). In the end, it should be noted that the sequence administration of GEM and OVs and the effects of GEM on the virus replication could be considered in chemovirotherapy.

### 3.2 Gemcitabine and immune checkpoint inhibitors

The approval of ipilimumab, an immune checkpoint inhibitor (ICI), for the treatment of melanoma in 2011 (26) revolutionized the field of cancer immunotherapy. Immune checkpoints are immune cell receptors, either stimulatory or inhibitory, that regulate immune homeostasis by balancing effective immunity and self-tolerance (27). The upregulation of some inhibitory receptors, including programmed cell death 1 (PD-1), PD-L1, cytotoxic T-lymphocyte associated protein 4 (CTLA-4), lymphocyte-activation gene 3 (LAG-3), T cell immunoreceptor with Ig and ITIM domains (TIGIT), T cell immunoglobulin and mucin domain-containing protein 3 (TIM3), and B and T lymphocyte attenuator (BTLA), on immune cells in malignancies led scientists to design monoclonal antibodies to block them, which are known as ICIs (28). Inhibitors of CTLA-4 (Ipilimumab), PD-L1 (Avelumab, Durvalumab, and Atezolimumab), and PD-1 (Cemiplimab, Pembrolizumab, and Nivolumab) have been approved by the FDA as therapeutic agents against various types of cancers (29).

Since it was noted that GEM treatment could increase the expression of PD-1 and PD-L1 (30, 31), accumulating evidence has shown that GEM + ICIs exert synergistic effects against cancer cells compared with monotherapy and conquer resistance to anti-immune checkpoint therapy (32, 33). For instance, Zheng et al. concluded that treatment with low-dose metronomic GEM (2 mg/kg) not only increased tumor-infiltrating T-cells and improved tumor vessel perfusion in a murine model of breast cancer, but also its pretreatment circumvented resistance to ICIs (34). In another study, Principe et al. investigated the effect of GEM and its combination with anti-PD-1 on the TME of pancreatic cancer. They found that long-term treatment of GEM reformed the immune landscape, characterized by an increase in MHC I, antigen presentation CCL and CXCL family chemokines, and expression of TNF-α, IFN-γ, TGFβ1, PD-L1, and PD-L2. The released substances changed the makeup of the tumor stroma, making cancer-associated fibroblasts (CAFs) resistant to GEM and increasing the production of TGFβ1. The GEM + anti-PD-1 treatment did not affect the disease progression of mouse PDAC in transgenic models, unless the mice additionally experienced genetic or pharmacologic elimination of TGFβ signaling. When TGFβ signaling is lacking, combining gemcitabine with anti-PD-1 treatment results in a strong response from CD8^+^ T-cells and a reduction in tumor size, significantly improving the overall survival rate (35). A study conducted by Ho et al. revealed that anti-cancer activities of GEM and anti-PD-1 are mediated by M1 macrophages and Th1 lymphocytes (36). To improve therapeutic efficacy by maximizing chemoimmunotherapy agents’ access to the TME and lessening systemic exposure, Wang et al. constructed injectable reactive oxygen species (ROS)-responsive hydrogel to deliver GEM and anti-PD-L1 (aPDL1-GEM@Gel) into the TME. Treatment with aPDL1-GEM@Gel not only significantly inhibited tumor growth in the B16F10 mouse melanoma tumor model, but also increased infiltration of CD4^+^ and CD8^+^ T-cells and induced systematic immune responses (37). It is worth noting that the combination of GEM and ICIs, such as ipilimumab, pembrolizumab, and nivolumab, is safe and tolerable in humans, according to clinical trial results (38–40).

### 3.3 Gemcitabine and CAR T-cells

CARs are recombinant receptors on T-cells, enabling them to recognize TAAs in a major histocompatibility complex (MHC)-independent manner. The extracellular domain of CARs is structurally constructed by single-chain antibody fragments (scFv) to recognize and bind to a particular protein on malignant cells, while their intracellular domain triggers signaling to activate CAR T-cells (41). According to the number and structure of costimulatory molecule(s) in the intracellular domain, five generations of CAR T-cells have been developed: the first generation CAR T-cells contain only a CD3ζ intracellular domain, whereas the second and third generations contain one and multiple costimulatory molecules, respectively, in their intracellular domain, such as CD28 and CD137 (41BB). Structurally, the fourth generation of CAR T-cells resembles the second generation, but they can produce a cytokine, such as IL-2 or IFN-γ. The fifth generation is also similar to the fourth one but they have an intracellular domain of a cytokine receptor instead of a cytokine expression inducer (42). In 2017, Tisagenlecleucel (Kymriah) was approved by the FDA as the first CAR T-cell for the treatment of acute lymphoblastic leukemia (AML) (43).

There are some reasons to combine CAR T-cells with chemotherapy: 1) chemotherapy can act as bridging therapy to cover treatment intervals for preventing disease progression, 2) chemotherapy creates an optimal condition for CAR T-cell therapy, such as acting as lymphodepleting regimen, 3) chemotherapy can act as adjuvant/neoadjuvant for CAR T-cell therapy, and 4) chemotherapy increases T-cell trafficking to tumors (44). Regarding GEM, a phase I/II clinical trial using CD-19-targeted third-generation CAR T-cell concluded that GEM-treated patients (3 out of 5) achieved complete response, the disappearance of all signs of cancer, following CAR T-cell administration. They explained that GEM improves the effectiveness of CAR T-cell therapy by increasing NK cells and CD8^+^ T-cells as well as reducing MDSCs within the TME (45). Moreover, GEM can enhance the killing ability of cytotoxic T-lymphocytes (CTLs) (46).

### 3.4 Gemcitabine and therapeutic vaccines

Although vaccines were considered as preventive agents against infectious diseases at first, development in this field led to the introduction of therapeutic ones against cancer. Therapeutic cancer vaccines aim to stimulate the adaptive immune system of patients against tumor antigens to control tumor growth (47). Although different platforms have been used in the design of therapeutic vaccines, including nucleic acid-based, protein/peptide-based, vector-based, and cellular-based (48), they are constructed by the combination of tumor antigens, either tumor-specific antigens (TSAs) or TAAs, and adjuvants (49–51). Efforts in vaccine research led to the approval of four prophylactic vaccines against human papillomavirus (HPV)-induced cervical cancers (Cervarix, Gardasil, Gardasil9, and Cecolin) and hepatitis B virus (HBV)-related hepatocarcinoma (52, 53), while Sipuleucel-T is the only approved therapeutic vaccine which is based on dendritic cells (DCs) for the treatment of prostate cancer (54).

Similar to other immunotherapeutic strategies, vaccines also showed synergistic effects against malignant cells in combination with GEM (55). Bauer et al. indicated that GEM remarkably suppresses the stimulated antibody titers and antigen-specific CD8^+^ T-cells, whereas it facilitates CD8^+^ T-cell recruitment into the tumors in DC-vaccinated mice. Importantly, they suggested that GEM timing regulates DC vaccination efficacy, so delayed GEM administration circumvents chemotherapy-induced suppression of B-cells and T-cells and exhibits better tumor control efficacy (56). Also, in the combinational regimens, GEM increases epitope spreading by inducing tumor lysis (57). The other mechanism by which GEM contributes to improving the therapeutic activities of cancer vaccines is its ability to deplete regulatory T-cells (Tregs) and MDSCs while enhancing the activity of vaccine-specific CTLs (58, 59). Additionally, the immunomodulatory function of GEM in combination with vaccines could be due to its inhibitory effects on DC-IL-10^+^ cells, resulting in enhanced vaccine-induced CD4^+^ and CD8^+^ T-cell responses (60).

## 4 Immunomodulatory roles of gemcitabine

**Figure 3:**
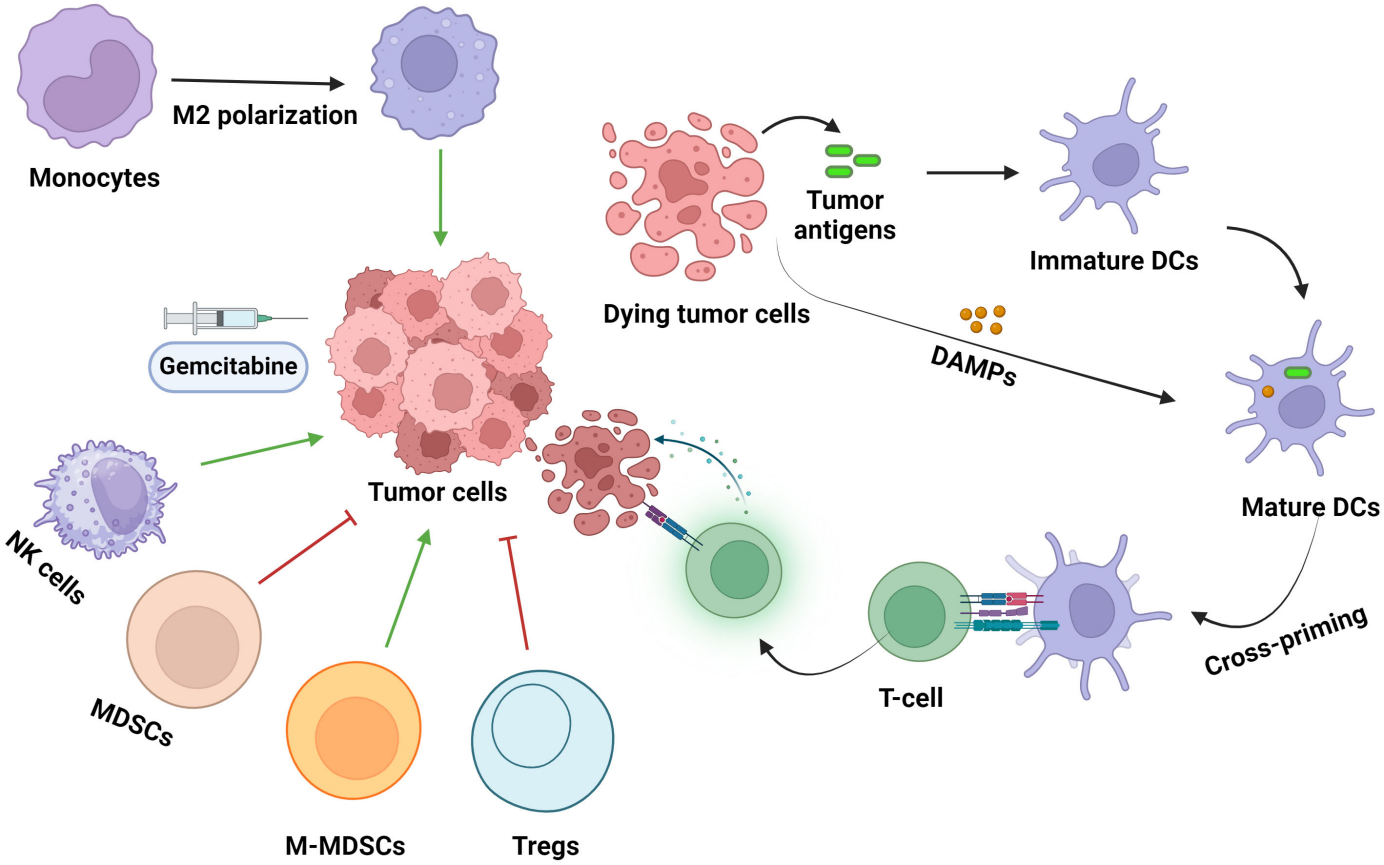
The effects of gemcitabine administration on immune cell populations within the TME. DC, dendritic cells; DAMPs, damage-associated molecular patterns; Tregs, regulatory T-cells; MDSCs, myeloid-derived suppressor cells; NK cells, natural killer cells. Green and red arrows indicate increased and decreased cell infiltration into the TME, respectively.

There is evidence depicting the immune-modifying functions of GEM and its widespread effects on immune cells, both circulating and local cells in the TME, and immune responses (**Figure 3**).

### 4.1 Natural killer (NK) cells

NK cells, 5-10% of peripheral blood lymphocytes, are generally classified into two subclasses: a subclass with immunomodulatory and cytokine production activity (CD56^bright^CD16^low/-^) and the other with cytotoxic activity (CD56^dim^CD16^+^) (61). Although their ability to kill tumor cells makes them promising candidates in cancer therapy, the hypoxic and immunosuppressive TME impede their potential (61). There is evidence that chemotherapy agents could promote the anti-cancer effects of NK cells by improving the recognition of tumor cells via NK cells within the TME, facilitating the infiltration of NK cells within the TME, and increasing the activity of NK cells via selective targeting of immunosuppressive cells (62). Regarding GEM, Zhang et al. designed a study to clarify its immunogenicity and mechanism of action both *in vitro* and *in vivo* models of lung cancer. They found that low-dose GEM treatment *in vitro* could enhance immunogenicity by increasing high mobility group box 1 (HMGB1) and calreticulin (CRT) exposure, two well-known DAMPs. Moreover, low-dose GEM treatment (30 mg/kg) induced the activation of NK cells in a mouse model of lung cancer via upregulating NKG2D ligands, including major histocompatibility complex class I polypeptide-related sequence A and B (MICA and MICB). The activated NK cells showed higher levels of IFN-γ production and more cytotoxicity against tumor cells, leading to sufficient inhibition of tumor growth. They concluded that the dose is a determining factor in inducing NK-mediated immune responses following GEM administration, so high doses of GEM impair the proliferation of NK cells (63). Okita et al. reported that the positive effect of GEM on the expression of NKG2D ligands and NK cell-mediated cytotoxicity is due to the activation of the ataxia telangiectasia mutated (ATM) and ATM- and Rad-3-related protein kinase (ATR) pathways following GEM-mediated DNA damage. Mechanistically, GEM treatment decreases the levels of miR-10b and miR-20a, which act as suppressors of NKG2D ligands (64). Another study revealed that the administration of GEM after R0 surgical resection of pancreatic cancer not only reduced the Cd11b^+^Gr1^low^F4/80^+^ subclass of MDSCs, but also increased the percentage of NK cells without affecting macrophage polarization and Tregs. Based on the increase in local recurrence and abrogate in prolonged survival after NK cell depletion, not CD8 T-cells, they concluded that the innate immune response is essential for GEM-mediated prevention of local relapse after surgery of pancreatic cancer (65).

### 4.2 Myeloid-derived suppressor cells (MDSCs)

It has been verified that MDSCs play crucial roles in the progression of tumor cells by facilitating tissue remodeling, providing immune evasion, supporting malignant cell survival, contributing to resistance to therapies, and promoting metastasis and angiogenesis (66). Due to the suppressive effect of MDSCs on T-cells, Bazargan et al. designed a study to assess the role of GEM, as a lymphodepleting agent, in enhancing the efficacy of adoptive cell therapy (ACT) in a mouse model of pancreatic cancer. Although GEM treatment (500 µg/mouse) remarkably reduced the population of T-cells, MDSCs, and overall immune cells, pretreatment of tumors preconditions the TME and enhances ACT efficacy in the treatment of cancer (67). To diminish the lymphodepletion activity of GEM and its adverse effect on other immune cells, selectively targeting MDSCs by encapsulating GEM within nanoparticles could be a reliable strategy (68). Encapsulated GEM could act as more effective than free GEM in the depletion of MDSCs, leading to a decrease in immunosuppressive molecules, such as PD-L1, and immunosuppressive cytokines, such as IL-6, IL-10, and TGF-β, as well as an increase in CTLs and pro-inflammatory cytokines, such as TNF-α and IFN-γ (69). In another study, Le et al. used GEM to inhibit the immunosuppressive function of MDSCs in 4T1 tumor-bearing mice according to two plans: EARLY GEM (once a week starting 5 days after tumor inoculation) and LATE GEM (a single dose at days 20-25). They showed that EARLY GEM not only inhibits MDSC accumulation in the spleen, but also reverses splenomegaly and delays tumor growth. Due to the inhibitory effects of MDSCs on CD8^+^ T-cells, they found that MDSC depletion following GEM treatment augmented T-cell expansion and enhanced IFN-γ production (70).

### 4.3 Regulatory T-cells (Tregs)

CD4^+^ Tregs are immunosuppressive cells within the TME, characterized by the transcription factor Forkhead box P3 (Foxp3) in the nucleus and CTLA-4, PD-1, and CD25 on the cell surface (71). These cells exert their immunosuppressive functions by converting ATP to adenosine which suppresses optimal T-cell activation, inhibiting activation of T-cells in a CTLA-4-mediated manner, secretion of factors, such as perforin and/or granzyme, for destroying effector cells, consumption of IL-2, and producing cytokines with immunosuppressive roles, such as TGF-β, IL-10, and IL-35 (72). Therefore, depletion of Tregs could be a reliable strategy to enhance anti-tumor immune responses. According to recent studies, not all Tregs cells are immunosuppressive and they can be classified into three classes: 1) Tregs with stabilizing and strong inhibitory functions, called effector Tregs, which are characterized by CD45RA^−^ and FoxP3^high^, 2) Tregs with weak inhibitory functions, called initial Tregs, which are identified by CD45RA^+^ and FoxP3^low^, and 3) Tregs that primarily secrete inflammatory cytokines, called non-Tregs, which are characterized by CD45RA^−^ and FoxP3^low^ (73). Several studies have used GEM to reduce the population of Tregs within the TME and circulation (58, 74, 75). For instance, Shevchenko et al. investigated the mechanisms by which Tregs accumulate in the tumor and how GEM could target Tregs in a mouse model of pancreatic cancer. Since a specific inhibitor of TGF-β receptor I kinase and a CCR4 antagonist failed to abrogate Treg accumulation, they concluded that local proliferation is the main mechanism of Treg accumulation. Additionally, they found that treatment with low-dose GEM (15 mg/kg) leads to a reduction in the numbers of proliferating Tregs and remarkably extends the survival of tumor-bearing mice (76). In another study, Eriksson et al. studied the immune profile using flow cytometry in GEM-receiving patients with pancreatic adenocarcinoma. They reported that the increased levels of Tregs, MDSCs, and TGF-β in patients with pancreatic adenocarcinoma were decreased after GEM administration (1000 mg/m^2^) once weekly for 3 weeks, while the T-cell population remained unchanged and NK cell population was reduced in most patients, indicating that NK cells are more sensitive than T-cells to GEM (31).

### 4.4 Dendritic cells (DCs) and T-cells

Due to their ability to coordinate both innate and adaptive immune responses, DCs play crucial roles in response to tumor cells. The production of growth factors and protective cytokines, such as IL-6 and IL-12, in response to danger signals signifies their participation in innate responses, while DCs contribute to adaptive responses through the presentation of antigens on MHC I and MHC II to naïve T-cells to generate effector anti-tumor cells (77). However, the immunosuppressive TME could impair the function of DCs and develop indoleamine 2,3‐dioxygenase (IDO)-, ICOSL-, and PD-L1-expressing DCs or promote the expansion of Tregs, leading to tumor growth and immune evasion (78). On the other hand, cytotoxic T lymphocytes (CTLs) play prominent roles in killing malignant cells by different mechanisms, including activation of other immune cells, triggering death receptor pathways, and production of cytotoxic granules (79). There is evidence that GEM could modulate cancer immune responses by affecting DCs and CTLs. For instance, an assessment of the immune profile in GEM-treated patients with advanced pancreatic cancer revealed that GEM treatment (1000 mg/m^2^ for 3 weeks) could significantly increase the percentage of myeloid DCs (CD11c^+^) and plasmacytoid DCs (CD123^+^) (80). Pei et al. found that the released heat shock protein 70 (Hsp70) from the dead cells following GEM treatment is the mechanism by which GEM induces DC maturation. Mechanistically, Hsp70 mediates DC maturation and activation in the tumor milieu by transporting peptide antigens into DCs. They also indicated that GEM-treated medium + DCs could stimulate specific CTLs against pancreatic tumor cells. Therefore, the released DAMPs and antigens following GEM-induced immunogenic cell death (ICD) could promote DC maturation and CTL responses (81). In addition to the release of danger signals, GEM contributes to CTL-mediated killing of tumor cells by triggering FasL cytotoxicity. Pei et al. showed that GEM increases the sensitivity of pancreatic cancer cells to CTL anti-tumor responses in the Fas-dependent pathway. Indeed, GEM increases the expression of Fas on the tumor cells, even at low doses, and the interaction between Fas on the tumor cells and FasL on the CTLs leads to the killing of malignant cells (82). Also, the ability of GEM to upregulate MHC I by tumor cells and enhance their stability on the cell surface is another mechanism by which GEM promotes CTL functions (46, 83). Therefore, GEM affects CTL immune responses using different mechanisms.

## 5 Immunosuppressive roles of gemcitabine

Despite great evidence for the immunomodulatory roles of GEM, studies show that GEM could promote an immunosuppressive niche within the TME. For instance, Deshmukh et al. found that GEM creates an immunosuppressive TME in GEM-treated mice with an orthotopic human pancreatic tumor xenograft by inducing growth, infiltration, and polarization of macrophages toward tumor-associated macrophages (TAMs), characterized by upregulation of TGF-β1 and arginase-1 (84). Similarly, Bulle et al. showed that GEM treatment (50 mg/kg) shifts macrophage polarization toward the M2 population in EMT-high tumors, in which tumor metabolic profile acts as a favorable element in M2 polarization. M2 macrophages rely on fatty acid oxidation for ATP production and have an intact tricarboxylic acid (TCA) cycle, whereas M1 subtypes produce ATP by glycolysis and show a defect in the TCA cycle (85). It was reported that IL-8 is the determining factor in inducing macrophage growth and directing them to the TME, while its role in M2 polarization was unclear (84). Mechanistically, IL-8 binds to CXCR1 and CXCR2 on macrophages, which triggers NF-ĸB activity, leading to the upregulation of MMPs and facilitating their invasion (86, 87). Moreover, there is evidence that the TME macrophages act as scavengers that metabolize and inactivate GEM, whereas TAM depletion sensitizes tumor cells to GEM (88). It is worth noting that macrophages within the TME are divided into two classes: macrophages with anti-tumor activities, called M1 phenotypes, which are identified by the expression/secretion of CD64, CD86, IL-12, ROS, and inducible nitric oxide synthase (iNOS), and macrophages with pro-tumorigenic activities, called M2 phenotypes, which are characterized by the expression/secretion of CD163, CD206, arginase-1 (ARG-1), and TGF-β (89–91). M2 macrophages contribute to tumor development by promoting tumor angiogenesis, metastasis, and drug resistance and inhibiting T-cell mediated immune responses against tumor cells (92). In addition to the roles of macrophages in GEM-mediated immunosuppression, it has been shown that repeated GEM treatment (60 mg/kg) generates an immunosuppressive TME by favoring differentiation of immunosuppressive Ly6C^high^ monocytic MDSC (M-MDSC) cells through overexpression of GM-CSF, a cytokine for differentiation of M-MDSCs, and efferocytosis, phagocytosis of apoptotic malignant cells. The hyperproduction of mitochondria reactive oxygen species (mtROS), as well as NF-ĸB activation, are responsible for GM-CSF upregulation in the TME following GEM treatment. Furthermore, GEM could induce resistance to T cell-mediated killing by increasing lipid metabolism and lipid droplets in residual tumor cells (93). In a mechanical overview, the oxidization of accumulated lipids by ROS hinders antigen-mediated cross-presentation and prevents T-cell stimulation (94). Additionally, GEM treatment (20 mg/kg) stimulates the production of CCL2 in the tumor cells which recruits M-MDSC to the tumor niche, and CCL2 receptor antagonists, such as RS 504393, could block M-MDSC recruitment (95).

## 6 Conclusion and future perspectives

Chemotherapy is a gold standard of cancer treatment due to its cytotoxic effects against replicating cells, however, there is evidence that chemotherapeutic agents can affect immune cells and responses. These characteristics caused the advent of the chemoimmunotherapy approach to the cancer therapy field in which traditional chemotherapy is combined with immunotherapy. Regarding GEM, its combination with oncolytic viruses, ICIs, CAR T-cells, and therapeutic vaccines improved their therapeutic efficacy and augmented immune responses. GEM could positively modulate immune responses within the TME by activating NK cells, depleting MDSCs and Tregs, eliciting immunogenic cell death, and releasing antigens and DAMPs, which maturate and activate DCs to stimulate CTLs. Moreover, GEM could create an immunosuppressive TME by directing the differentiation of macrophages toward M2 ones and increasing the recruitment of immunosuppressive cells, such as M-MDSCs. The immunomodulatory roles of GEM faced some considerations. Firstly, a high dose of GEM not only kills malignant cells, but also negatively affects immunogenicity and immune cells. In contrast to high-dose GEM which inhibits anti-tumor immune cells, GEM at low-dose could activate immunomodulatory immune cells and increase their proliferation. At a low dose, GEM suppresses tumor growth with fewer side effects. Moreover, a low dose of GEM reduces immunosuppressive cells, such as MDSCs and Tregs, whereas its high dose significantly decreases lymphocyte cells. The sufficient immune cells within the TME and peripheral circulation following treatment with a low-dose GEM ensures its effectiveness in the combinational regimen with immunotherapy agents. One of the solutions to hamper the adverse side effects of GEM is using nano-delivery systems to target specific immune cells precisely. Nano-based delivery systems also increase the bioavailability and solubility of GEM and could re-educate the TME to augment immune responses. Secondly, administration timing is crucial for optimizing GEM combination with other therapeutic agents, especially other immunotherapy cell strategies. Early treatment with GEM preconditions the TME to boost anti-tumor immune responses. Preconditioning with GEM not only depletes immunosuppressive cells, but also induces ICD and promotes the release of DAMPs which initiate and prolong immune responses. Therefore, addressing these challenges and overcoming the immunosuppressive functions, GEM could be a reliable chemoimmunotherapy agent, both as monotherapy or in combinational regimens, for the treatment of cancer.
